# From knowledge to action: participant stories of a population health intervention to reduce gender violence and HIV in three southern African countries

**DOI:** 10.1080/09540121.2014.931560

**Published:** 2014-07-03

**Authors:** Mary Cameron, Anne Cockcroft, Grace Wanjiru Waichigo, Nobantu Marokoane, Ditiro Laetsang, Neil Andersson

**Affiliations:** ^a^ CIETcanada, Ottawa, ON, Canada; ^b^ CIET Trust Botswana, Gaborone, Botswana; ^c^ CIET Trust, Johannesburg, South Africa; ^d^ Centro de Investigación de Enfermedades Tropicales (CIET), Universidad Autónoma de Guerrero, Acapulco, Mexico; ^e^ CIET/PRAM, Department of Family Medicine, McGill University, Montreal, Canada

**Keywords:** implementation research, HIV, sexual violence, sexual behaviour, intervention studies, process evaluation

## Abstract

This paper describes implementation research of an intervention in a complex HIV prevention randomised trial in southern Africa. Researchers collected stories of change attributed by 106 community members to an audio-drama edutainment intervention in 41 sites in Botswana, Namibia and Swaziland. The team analysed themes in the stories following a behaviour change model of conscious knowledge, attitudes, subjective norms, intention to change, agency, discussion and action (CASCADA). Storytellers attributed positive changes to the intervention in the areas of gender violence, multiple sexual partners, transactional and intergenerational sex and condom use. Their stories illustrate each of the steps in the CASCADA behaviour change model. As well as supporting an enabling environment for other interventions in the trial, the audio-drama also helped some participants to make personal changes. Collecting and discussing the stories were encouraging for the trial fieldworkers. Documenting the experiences of participants and framing the analysis of stories in an explicit behaviour change model allowed us to reflect on potential mechanisms and pathways through which the intervention impacts on individuals and communities. It helped in the design of the quantitative instruments to measure intermediate outcomes of the trial.

## Background

Gender violence is a serious public health concern worldwide (Alhabib, Nur, & Jones, 2010; Ellsberg, Jansen, Heise, Watts, & Garcia-Moreno, 2008; Garcia-Moreno & Watts, 2011) and an important driver of the HIV epidemic in southern Africa (Andersson, Cockcroft, & Shea, 2008; Campbell et al., 2008; Jewkes, Dunkle, Nduna, & Shai, 2010; Townsend et al., 2011; Watts et al., 2010). The prevention of gender violence and HIV requires complex interventions to address the underlying and interacting social, cultural, economic, political and biological mechanisms.

Complex interventions have multiple interacting components, and their evaluation is a growing area of investigation (Sullivan, 2009). Unlike clinical trials of efficacy of a single pharmaceutical agent, it can be difficult to pinpoint the components of complex population health interventions that are working or not working, and why (Campbell et al., 2000). There is a need for a good theoretical understanding of how an intervention causes change, so weak links in the causal chain can be identified and strengthened; a process of evaluation to identify implementation problems; and measurement of unexpected consequences as well as the primary outcome (Campbell et al., 2000; Craig et al., 2008).

Uncovering causal pathways in complex interventions implies recognition of intermediate outcomes. Measuring only a single endpoint might divert attention from important contextual factors, feedback loops, users' experiences and learning processes, and individualised long-term outcomes (Bonell, Fletcher, Morton, Lorenc, & Moore, 2012; Paterson, Baarts, Launso, & Verhoef, 2009). Causal models allow consideration of a broader range of outcomes or indicators of change (Hardeman et al., 2005; Paterson et al., 2009).

Two recent randomised controlled trials in South Africa tested complex interventions to reduce gender violence and HIV infection with encouraging intermediate outcomes (Jewkes et al., 2008; Pronyk et al., 2006), including reduction in sexually transmitted infections, transactional sex, problem drinking, intimate partner violence (IPV), as well as improved socio-economic well-being and attitudes to gender disparities (Jewkes et al., 2008; Pronyk et al, 2006).

We recently implemented a cluster randomised controlled trial (CRCT) testing community-based interventions to reduce HIV infection in Botswana, Namibia and Swaziland (CIET, 2012; ISRCTN, 2008). We hypothesised that reducing gender violence and choice disability – the inability to act on choices to protect oneself from HIV infection (Andersson, 2006) – will reduce new HIV infections.

One intervention in this trial is an educational audio docudrama series, *Beyond Victims and Villains* (BVV). Based on findings from studies in schools and households in the region (Andersson & Ho-Foster, 2008; Andersson, Ho-Foster, Mitchell, Scheepers, & Goldstein, 2007; Andersson et al., 2004, 2012), the eight episodes present and discuss with community groups evidence about violence against women, sexual violence, abuse of children, transactional sex, trans-generational sex, choice disability and HIV risk.

Botswana, Namibia and Swaziland all have high rates of HIV, particularly among young women. The 2008 baseline survey of the trial (CIET, 2012; ISRCTN, 2008) reported HIV prevalence among young women aged 15–29 years of 22.9% in Botswana, 9.1% in Namibia and 26.1% in Swaziland (Andersson & Cockcroft, 2012). Four structural factors – lower education, income disparity with partner, poverty (insufficient food) and experience of IPV – were associated with a higher HIV prevalence (Andersson & Cockcroft, 2012).

In addition to quantitative measurement of the trial impact, to supplement our understanding of how the BVV intervention affected participants, we used the qualitative most significant change (MSC) technique (Dart & Davis, 2003; Davies & Dart, 2005) to collect and analyse stories from community members who participated in the BVV discussion groups. This paper describes the findings from the narrative approach, in the light of a behaviour change model.

## Methods

### The BVV intervention

The trial randomised 77 communities in Botswana, Namibia and Swaziland in a factorial design to receive one, two or three interventions or no intervention. Some 41 communities received the BVV intervention alone or in combination. Trained fieldworkers and community facilitators convened community groups to listen to and discuss the eight BVV episodes. The intervention was the *offer* of the BVV programme, aiming to convene separate groups covering older and younger men and older and younger women. Facilitators encouraged participants to discuss topics with family and friends between the weekly meetings.

Design of the BVV intervention drew on a model of behaviour change that extends the knowledge, attitudes and practices (KAP) model of behaviour change. The acronym CASCADA describes a partial order of intermediate outcomes through conscious knowledge, attitudes, subjective norms and positive deviation from these, the intention to change, the agency (individual and collective) to make change, discussion of possible action and finally action to change (Andersson, 2011; Andersson & Ledogar, 2008). The CASCADA expanded model responds to recognised limitations of the KAP model (Bentler & Speckart, 1979; Baranowski, Cullen, Nicklas, Thompson & Baranowski, 2003). We have used the CASCADA model in a trial of an intervention to increase vaccination uptake in Pakistan (Andersson et al., 2005, 2009) and in a cross-sectional study in southern Africa (Mitchell, Cockcroft, Lamothe, & Andersson, 2010).

The eight BVV episodes address components of the CASCADA model. Each BVV session addresses several CASCADA components. All sessions include discussion of the contents of the episode and consider possible actions. Early episodes of the docudrama and their discussion focus more on conscious knowledge and individual attitudes, while later episodes build on this to stimulate participants to confront attitudes and social norms, to feel able to make changes and to plan and discuss actions.

### Qualitative evaluation of BVV

We used the MSC technique to evaluate implementation of the intervention (Dart & Davies, 2003; Davies & Dart, 2005). The MSC technique involves collecting stories of change from programme participants, allowing participants to describe changes most meaningful to them, which may not otherwise be captured. It can document both anticipated and unanticipated effects.

Between November 2011 and January 2012, field staff (six women and four men) collected stories of change from BVV participants and community facilitators in BVV communities. They identified storytellers guided by the community facilitator, their own knowledge of participants and participants themselves. This selection provided a range of perspectives but did not attempt to measure the average or typical experience.

After verbal consent, the interviewer invited the interviewee to tell him/her a story: “Please tell me a story describing what you think is the most significant change in your life as a result of your involvement in BVV”. Interviewees were free to tell stories of negative changes or to say that they had not experienced any change. The interviewers took notes as the storytellers spoke and then read back their notes to check they had captured the story correctly. The field teams from all three countries later met to discuss the stories.

A researcher not involved in story collection entered the stories, translated into English, into Text Analysis Mark Up System (TAMS) Analyzer that supports coding, searching and summarising qualitative data (Weinstein, 2012). We analysed the text using codes representing each step in the CASCADA model to identify themes of change in the stories, and codes for the corresponding storytellers' age and sex.

## Results

Most (85/108) participants and facilitators reported some changes they attributed to their participation in the BVV intervention. Common areas of change included gender violence, women's rights, multiple concurrent partners, transactional and trans-generational sex, condom use and alcohol use. Some described changes only in the early steps of CASCADA, such as knowledge and attitudes. Others told of changes in later steps in the sequence, such as feelings of agency, discussion of change and actual change; they sometimes described profound changes in behaviour and life situation.

### Conscious knowledge

BVV episodes shared findings from recent surveys in the country and region to increase conscious knowledge about issues such as the frequency of HIV, gender violence and transactional sex. Storytellers described how the sessions filled gaps in their knowledge and how they had shared the knowledge with others or how it had helped them to take action to improve their lives:

I learnt that it is not good to have multiple sexual partners. Not only does it put you at risk of contracting HIV, it also means that men lose respect for you if they know that you “jump” around. (Namibia, 25-year-old woman)

I never knew much about condoms…. Now I know almost everything … this has helped me to become able to plan and resolve issues in my family. (Swaziland, 48-year-old man)

### Attitudes

The BVV sessions stimulated discussion of prevailing attitudes that supported gender inequality and acceptance of gender violence and risky sexual behaviours. Confronting these attitudes in discussions following the BVV episodes helped some people to question and change their own attitudes:

I used to have many partners and to me having many partners was something to write home about … I realised that behaviour was not going to take me anywhere. (Botswana, 22-year-old man)

### Subjective norms

Facilitators encouraged participants to recognise and critique the norms widely held in their community that can perpetuate acceptance of or indifference to gender discrimination and gender violence, and lead to increased risks of HIV.

Both men and women described pressure to conform to “normal” behaviour in their communities and peer groups, and how they had managed to break away from this:

We were brought up traditionally … to believe that you do whatever your husband demands … What we did not know was … our rights as women were not put into consideration. (Namibia, 45-year-old woman)

My friends and I use to compete over how many ladies we had sex with. The more ladies … the more man you are. But after I attended [BVV], I realise … We are putting our lives at risk just to show that we are men … Real men are those who take responsibilities for their lives and others. (Namibia, 27-year-old man)

Some young women described how norms around them were in favour of trans-generational sex:

I used to think sugar daddies were the “in thing” that youth should do … I was thinking of getting one before taking part in BVV sessions and peer pressure possibly could have accelerated my thinking. (Botswana, 23-year-old woman)

### Intention to change

At the end of each BVV session, the facilitator asked the group to consider how they could change things in their community or in their own lives.

Some admitted that they had not yet changed their behaviour but did intend to change:

I would really like to change and stop beating my wife but the problem is that she is full of nonsense: she will push me until I beat her up. (Botswana, 43-year-old man)

Some described how the BVV programme triggered a *turning point* when they realised the risks they faced and were motivated to make a change:

My husband is very abusive … he comes home drunk and demands sex … I can't live this way anymore. I am just waiting for my daughter to finish grade 12 this year and then we are moving back to my parents. (Namibia, 45-year-old woman)

### Agency

Some storytellers, especially women, described how they felt unable to control aspects of their lives:

I used to date older men … They gave me money and I gave them sex but not willingly as I had no choice. (Botswana, 39-year-old female)

Others told stories about feeling empowered to make changes after attending the BVV sessions:

As a woman, one never had a “voice” in marriage, whatever the husband says goes … I am applying what I have learned … I know my rights and will not allow anyone to control my life … I feel so empowered. (Namibia, 57-year-old woman)

### Discussion before action

In the CASCADA model, the immediate precursor to change is discussion. The BVV process deliberately stimulated discussion, both within the BVV group and with family and friends in the community.

Women in particular described how sharing personal stories and speaking openly in the BVV discussion groups encouraged them to voice their opinions and express their feelings elsewhere, and to seek change in their lives:

I had a problem with opening up about these personal issues, but after BVV, I share with people who come to me with similar problems. (Swaziland, 45-year-old woman)

Young men and women said they felt more comfortable talking openly about sex with their boyfriend or girlfriend, making it easier to refuse unwanted sex and negotiate condom use:

I talked to my boyfriend about what I have learned. We talk openly about safe sex. We decided that because we love each other … we should make it our priority to protect each other. (Namibia, 25-year-old woman)

### Action

After each BVV episode, the group discussed possible actions to address the issues raised, both individually and in their community.

Storytellers described how BVV enabled them to take action and make changes in their lives:

I was dating this guy who abused me emotionally … I started taking part in BVV sessions and I realized that I could leave him. I did that, and now my life has improved. I am working [and] saving money to buy stock for a tuckshop. (Botswana, 40-year-old woman)

Spending time at the tavern tempted me to hook up with drunken girls … I would have sex with these girls, sometimes unsafe sex. I knew that having multiple sexual partners was wrong, but hearing it from BVV tempted me to change. For the past 2 months, I haven't been to the tavern. Even if I do go there, I leave early, with no one by my side. (Swaziland, 21-year-old male)

### Sequence of change

Some participants described a sequence or series of changes from conscious knowledge, through multiple intermediate steps to action:

There is a lot of alcohol abuse and promiscuity among us, the youth. We all do it [norms]. I used to drink a lot and was having sex with more than one guy … After the BVV sessions I realised the danger I was exposing myself to [knowledge] … I didn't use a condom all the time and I didn't know my status. Because [of] the serious discussions we had at the BVV sessions [discussion], I managed to get the courage [agency] to go and test [action], and I found out I was negative … Now I always use a condom and I stopped sleeping around [action] … I am never putting myself at risk of infection again. (Botswana, 25-year-old woman)

### No change

Some people approached by interviewers reported no change from participation in the BVV programme. Most of them had recently begun the programme, and mentioned it was “too early” for change.

## Discussion

Some authors have criticised the KAP model of behaviour change (Bentler & Speckart, 1979) for failing to acknowledge the complexity of behaviour change. Baranowski et al. (2003) concluded that “the concepts of knowledge and attitude [in the KAP model] need to be more clearly specified conceptually and related to other variables within an overall process of change”. Awareness of risk is not enough to change behaviour (Anderson, Beutel, & Maughan-Brown, 2007; MacIntyre, Rutenberg, Brown, & Karim, 2004). By including a number of intermediate steps, the CASCADA model helps to overcome some of the concerns with the KAP approach.

Hardeman et al. (2005) outlined how causal models can contribute to development and evaluation of complex interventions. The development of BVV took into account the CASCADA model of intermediate outcomes. The episodes of BVV cover relevant knowledge, and explicitly address attitudes and social norms, while discussions help participants to plan changes.

Young women's involvement in transactional and trans-generational sex provides an example of the complexity of behaviour change. Women in southern Africa are involved in transactional and trans-generational sex for many reasons (Jewkes, Dunkle, Nduna, & Shai, 2012; Jewkes, Morrell, Sikweyiya, Dunkle, & Penn-Kekana, 2012). They are often well aware of the risks (Cockcroft et al., 2010). Economic needs, social norms and peer pressure play a part (Leclerc-Madlala, 2003, 2008). The stories told by young women BVV participants highlighted their individual reasons for reducing trans-generational and transactional sex.

The stories describing changes in intermediate outcomes in the CASCADA model shed light on how the BVV programme might reduce gender violence and HIV risk. Stories illustrated each element of the model, some indicating a series of changes leading to action. We cannot conclude from this study which of the CASCADA steps are more effectively addressed by BVV; this requires further investigation.

The BVV intervention aimed to create an enabling environment in communities to enhance the effect of a structural intervention to empower young women. The MSC stories showed that, for some people, BVV could also lead to profound personal changes, with increases in conscious knowledge leading to action through a series of intermediate steps. This enriched our understanding of the BVV programme and how we might use it in the future.

## Limitations

The MSC technique may overemphasise positive changes as storytellers asked about the MSC in their lives might be more inclined to report positive changes. The technique does not claim to produce an objective and representative description of the average programme effect; it indicates what changes *can* happen that are most meaningful to the participants themselves.

Selection of the storytellers was not random. The BVV facilitators probably suggested as storytellers people who had participated actively in the BVV sessions. Thus the stories reflect what *can* change as a result of participation, not what happens on average.

The reaction of the storytellers is likely to have been influenced by the interviewer. All interviewers were from the country concerned and spoke the language of the storytellers. Generally, male and female interviewers gathered stories from people of their own sex and similar age group. We did not document the education of each storyteller but it is likely that the interviewers were more educated.

The CASCADA model is work in progress. We have found it useful in a range of circumstances, but we do not suggest that behaviour change follows the partial order in a predictable way. The stories we collected here show that CASCADA steps can help to describe behaviour change. More work is needed to understand the relative importance of different elements of CASCADA in different circumstances and among different population groups.

## Conclusions

CRCTs tackling complex population health issues typically involve multiple interventions with many intermediate outcomes. Qualitative techniques for exploring outcomes from the standpoint of participants can usefully supplement quantitative impact measurement. Behaviour change models like CASCADA could help in the development and evaluation of complex interventions, providing a framework for understanding the pathways through which the intervention might function and for designing impact assessment instruments and procedures.
